# Supplementary material for: Chemical modifications of adenine base editor mRNA and guide RNA expand its application scope
Source: Nat Commun. 2020 Apr 24;11:1979. doi: 10.1038/s41467-020-15892-8 (PMC7181807; doi:10.1038/s41467-020-15892-8)
Supplement: Supplementary file 1 — Supplementary Information [file 41467_2020_15892_MOESM1_ESM.pdf]

Supplementary Information

Chemical modifications of adenine base editor mRNA and guide RNA expand its application scope

Jiang et al.

Supplementary Fig. 1

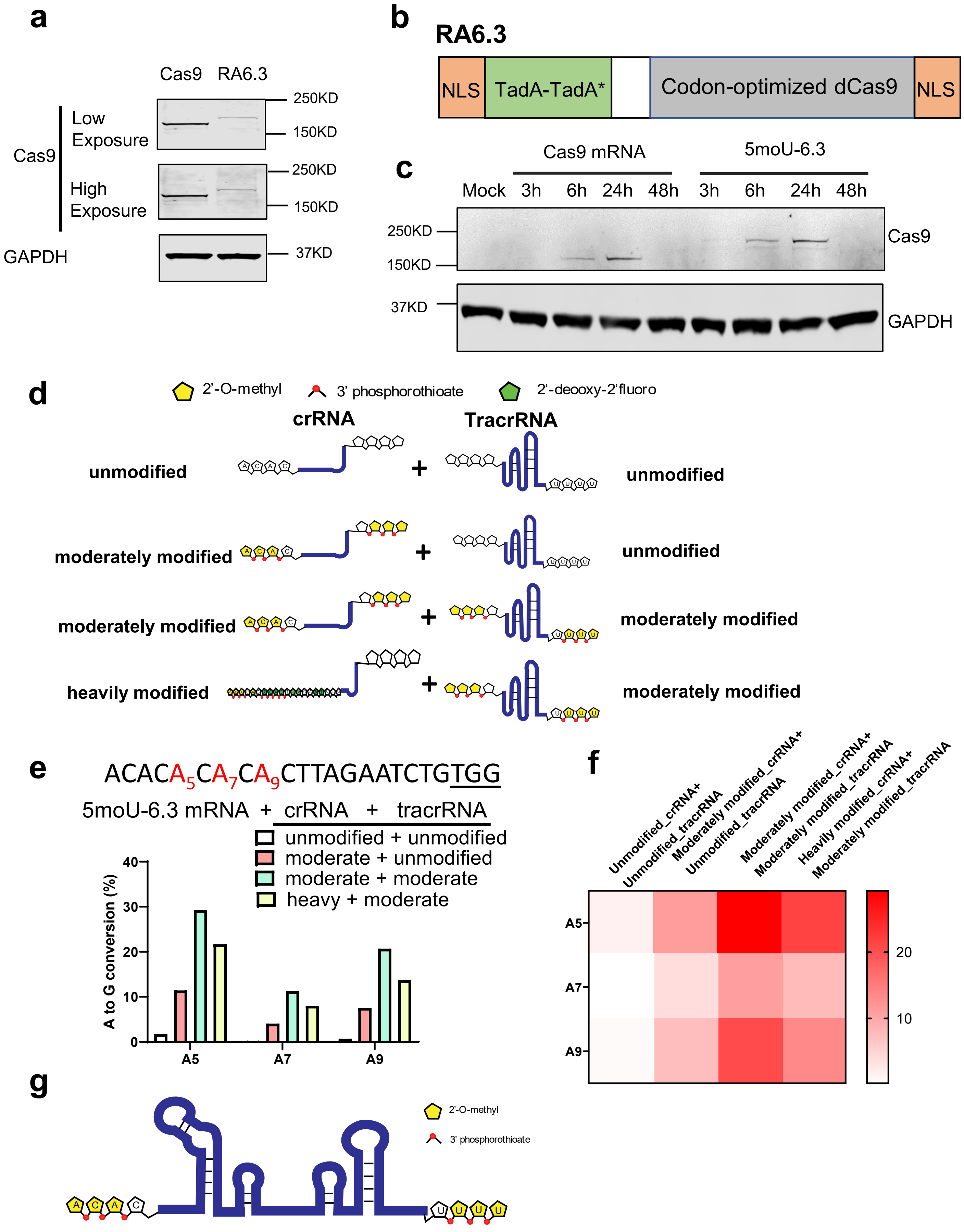

Supplementary Fig.1. a, The expression of Cas9 mRNA and in vitro transcribed RA6.3 mRNA in HEK293T cells. GAPDH as loading control. Experiments were done for twice, and one is shown. **b,** Schematic structure of RA6.3. NLS: nuclear localization signal. TadA* denotes the evolved TadA version 6.3[1]. **c,** Protein expression level at different time points in post-transfected HEK293T cells by western blot. Experiments were done for twice, and one is shown. **d,** Schematic structure of unmodified and differently modified tracrRNAs and crRNAs **e,** A-to-G conversion rates mediated by 5moU-6.3 with different combinations of tracrRNA and crRNA in HEK293T cells. The editable “A” substrate nucleotides are in red. PAM sequence is underlined. The data is based on one experiment. **f,** Heatmap of conversion rates shown in e. **g,** Schematic structure of moderately modified sgRNA. Source data are provided as a Source Data file for a, c, e, f.

Supplementary Fig. 2

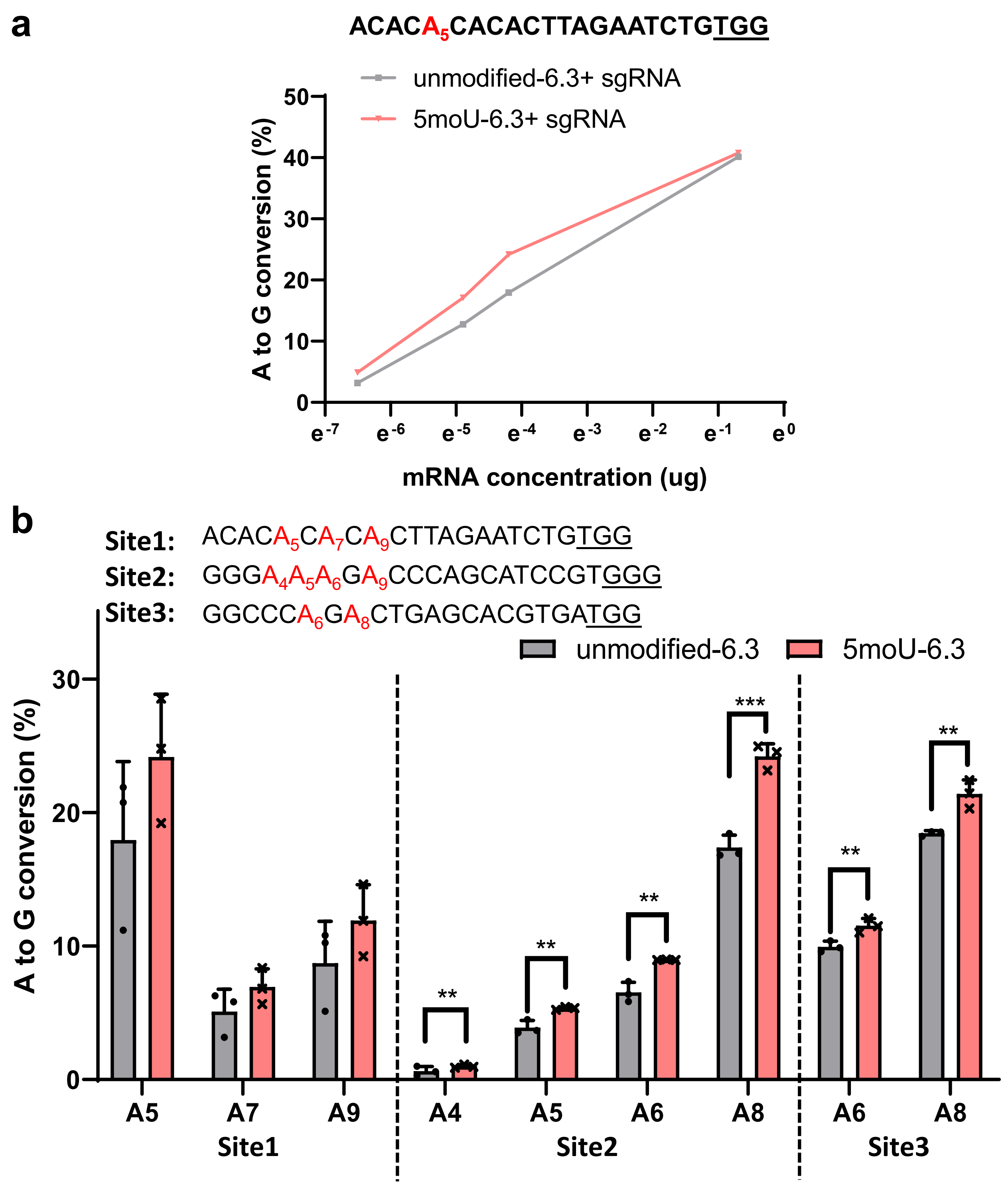

Supplementary Figure 2. a, Comparison of editing efficiency by unmodified (unmodified-6.3) and modified (5moU-6.3) ABE mRNA at different concentrations in HEK293T cells. Guide RNA is moderately modified sgRNA. A-to-G conversion rate represents the mean value of three independent experiments (n=3). The targeted editing site is highlighted in red. **b,** Comparison of editing efficiency by unmodified and modified ABE mRNA at the genomic sites shown in Fig1c, d, e in HEK293T cells. Guide RNAs are moderately modified sgRNA. Graphs show mean values. Data represent mean ±SD (n = 3 biologically independent samples). **, P= 0.0025, 0.0099, 0.0052; ***, P=0.0008; **, P=0.0162, 0.009 (two tailed t-test). mRNA concentration=0.015ug. Source data are provided as a Source Data file for a, b.

Supplementary Fig. 3

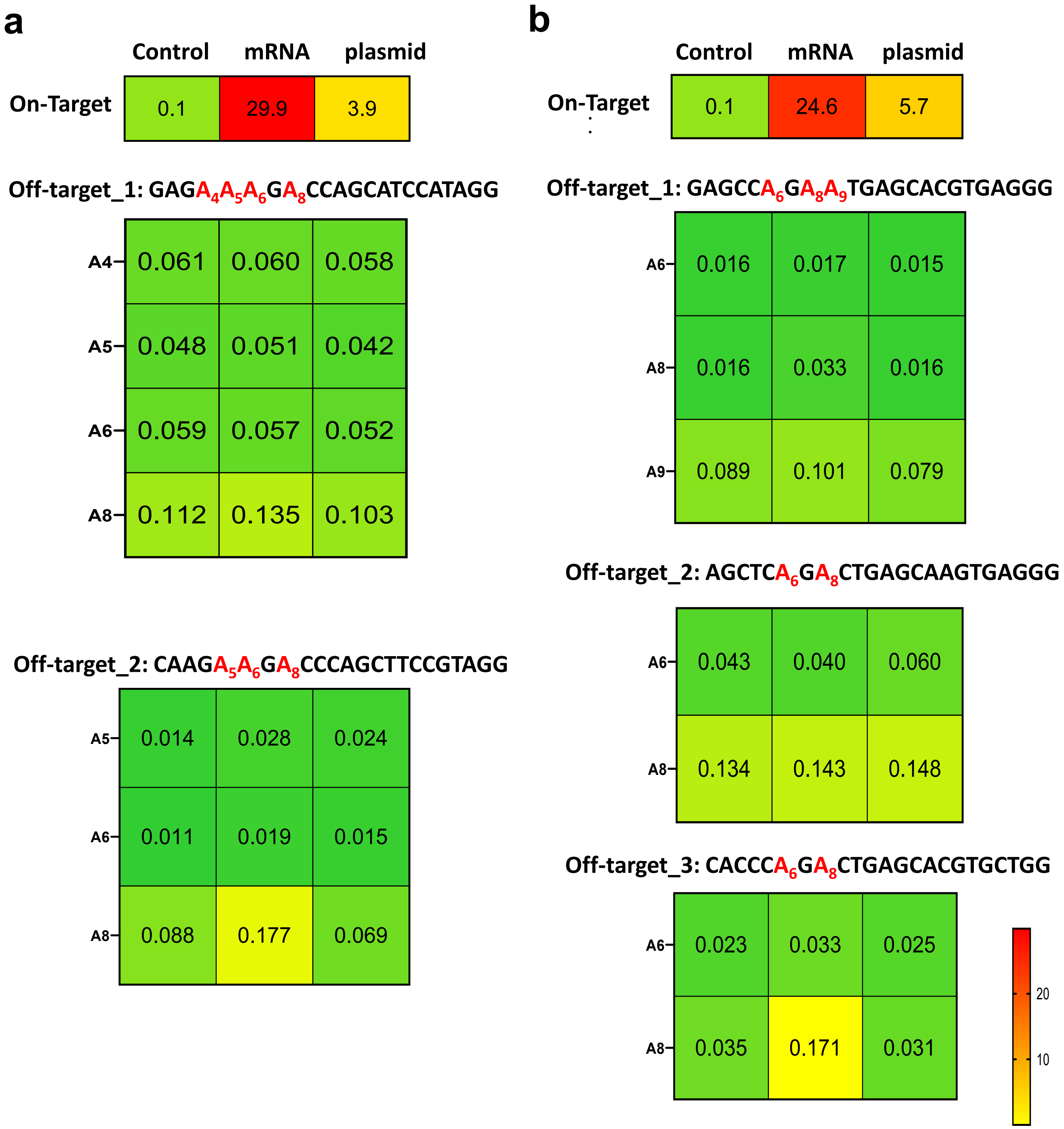

Supplementary Figure 3. a, Average triplicate A-to-G conversion rates (%) by RNA or DNA-encoded ABE at two top known off-target sites of guide RNA targeting the genomic site shown in Fig. 1d. The editing efficiency represents the mean value of three independent experiments. Off-target sites are as shown, and all the editable "A" sites are highlighted in red. **b,** Average triplicate A-to-G conversion rates (%) by RNA or DNA-encoded ABE at three top known off-target sites of guide RNA targeting the genomic site shown in Fig. 1e. The editing efficiency represents the mean value of three independent experiments. Off-target sites are as shown, and all the editable "A" sites are highlighted in red. Source data are provided as a Source Data file for a, b.

Supplementary Fig. 4

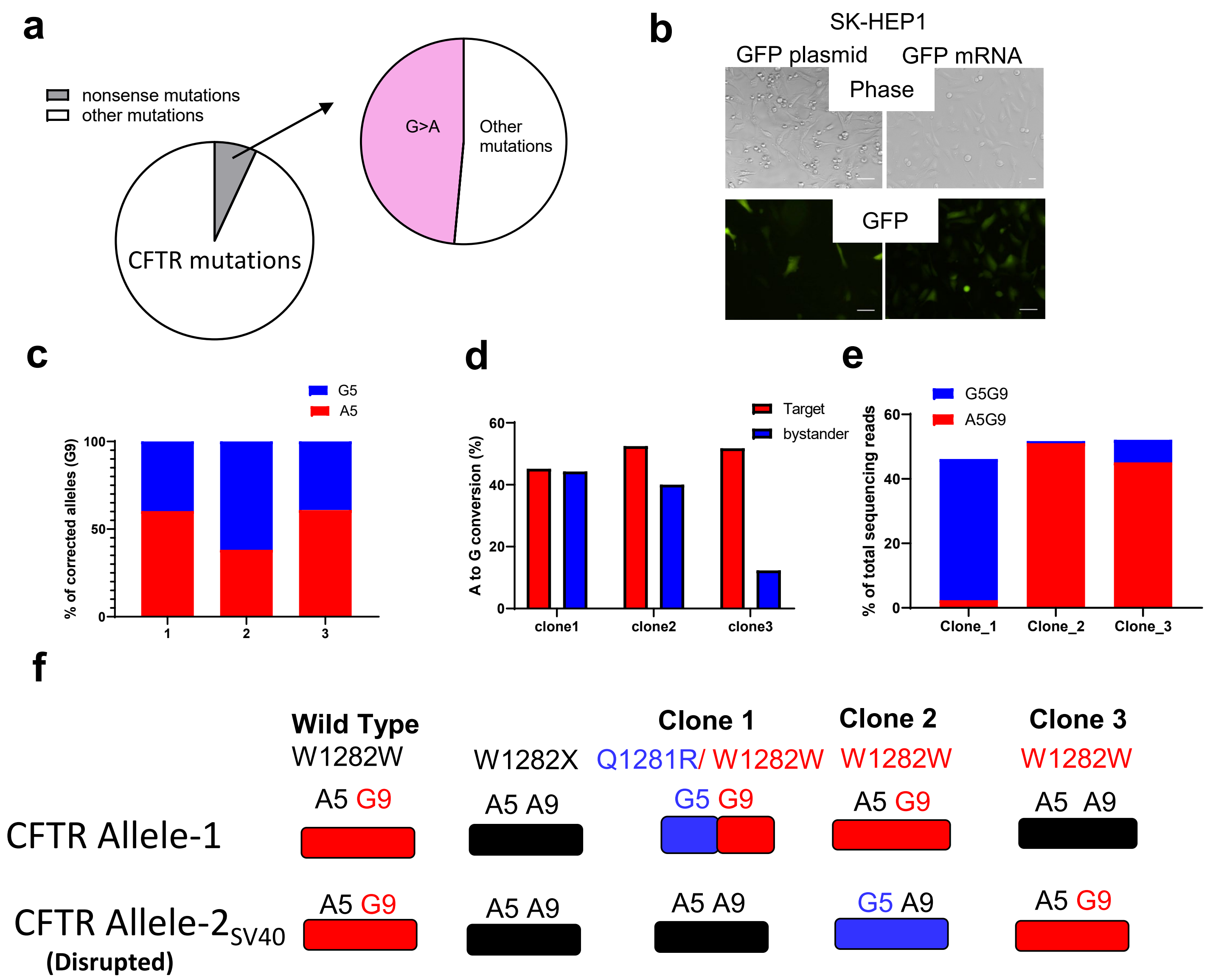

Supplementary Fig.4. a, Nonsense mutation frequency of all CF mutations (left graph), and G>A mutation frequency within all the CFTR nonsense mutations (right graph); concluded from CFTR2 database. **b,** Exogenous GFP DNA or mRNA expression in SK-HEP1 cells. 12hrs post electroporation, bright field (upper panel) and fluorescence images (lower panel) were taken. Scale bar=100 μm. **c,** The frequency of bystander editing in the allele that contained the targeted correction. Three biologically independent experiments. Blue = with bystander editing; Red = without bystander editing. **d,** A-to-G conversion rate at target and bystander sites of three single-cell clones. **e,** The frequency of bystander and target conversion at one *CFTR* allele in the three selected single-cell clones. Clone 3 is likely a mixed clone, but majority of the reads (~80%) have targeted editing at one allele without bystander editing. Blue = with bystander editing; Red = without bystander editing. **f,** The status of editable sites on the *CFTR* alleles in selected single-cell clones, as inferred from sequencing, and CFTR expression and activity experiments. A5/G5: bystander site. A9/G9: target site. SV40 incorporated allele (allele-2) cannot express protein (Disrupted). Source data are provided as a Source Data file for c, d, e.

Supplementary Fig. 5

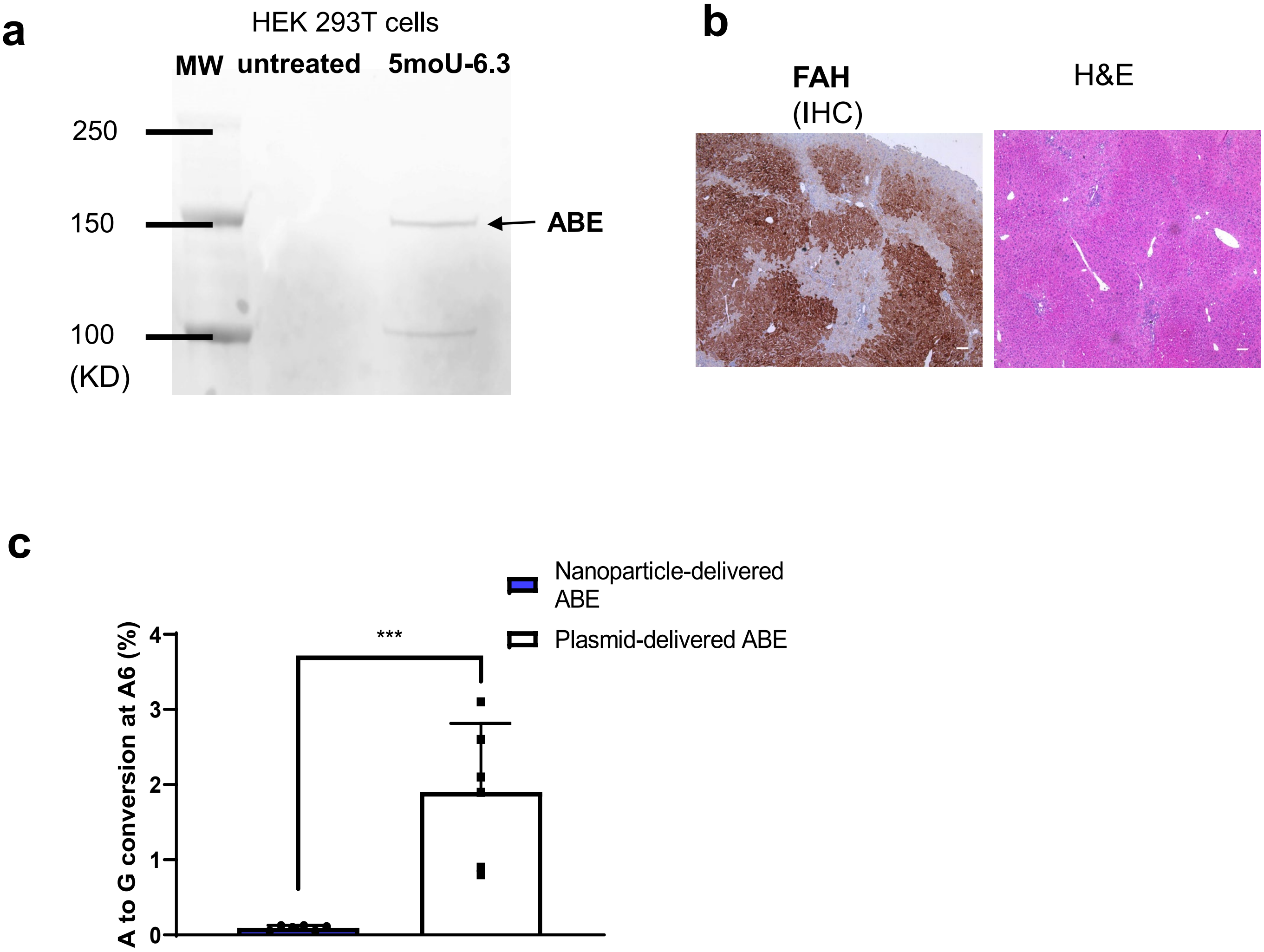

Supplementary Fig.5. a, Expression of LNP-delivered 5moU-6.3 in HEK293T cells. 4ul of ABE nanoparticle (0.9ug RA6.3 mRNA) was added to cell culture. After 6 hours, cell lysates were analyzed by western blot. Arrow indicates the full-length ABE protein band. **b,** Immunohistochemistry staining and Hematoxylin and Eosin staining (H&E) of mouse liver sections. Mouse was hydrodynamically injected with plasmids expressing RA6.3 and guide RNA (end point 48 days). Scale bar=100μm. **c,** A-to-G conversion rate at the bystander site (A6) of *Fah* locus by mRNA and DNA-expressing RA6.3 in vivo. Three liver samples (from different lobes) per mouse were collected and analyzed. ***, P=0.0006 (two tailed t-test). Data represent mean ±SD. Source data are provided as a Source Data file for a, c.

Reference:

1. Gaudelli, N. M. *et al.* Programmable base editing of A*T to G*C in genomic DNA without DNA cleavage. *Nature* **551**, 464-471, doi:10.1038/nature24644 (2017).

Supplementary Table

Description	Sequence
Unmodified Tracr RNA	rArArArCrArGrCrArUrArGrCrArArGrUrUr ArArArArUrArArGrGrCrUrArGrUrCrCrGrU rUrArUrCrArArCrUrUrGrArArArArArGrUr GrGrCrArCrCrGrArGrUrCrGrGrUrGrCrUr UrUrUrUrUrU
Medium-modified Tracr RNA	mrA*mrA*mrA*rCrArGrCrArUrArGrCrArArGrUrUr ArArArArUrArArGrGrCrUrArGrUrCrCrGrU rUrArUrCrArArCrUrUrGrArArArArArGrUr GrGrCrArCrCrGrArGrUrCrGrGrUrGrCrUr UrUrU*mrU*mrU*mrU
Unmodified crRNA	rArCrArCrArCrArCrArCrUrUrArGrArArUrCrUrGrGrUrUrUrArGrArGrCrUrArUrGrCr UrGrUrUrUrUrG
Medium-modified crRNA	mrA*mrC*mrA*rCrArCrArCrArCrUrUrArGrArArUrCrUrGrGrUrUrUrArGrArGrCrUr ArUrGrCrUrGrUrU*mrU*mrU*mrG
Heavily-modified crRNA	mA*mC*mA*rCrArC*rA*rC*/i2FA/*/i2FC/*/i2FU//i2FU/rA/i2FG//i2FA/rArU/i2FC/ /i2FU/rGrGrUrUrUrUrArGrArGrCrUrArUrGrCrUrGrUrUrUrUrG
Site1_libF	CTACACGACGCTCTTCCGATCTagcattacctgggagcctgttag
Site1_libR	AGACGTGTGCTCTTCCGATCTctcaaacttcagcgggcatcagaa
Site2_libF	CTACACGACGCTCTTCCGATCTcggcacgccccccttgtg
Site2_libR	AGACGTGTGCTCTTCCGATCTgggtctctagaccagcgtctt
Site3_libF	CTACACGACGCTCTTCCGATCTtctgctgcaagtaagcatgcatttg
Site3_libR	AGACGTGTGCTCTTCCGATCTaggaaaagctgtcctgcgac
Deepsequence_2nd_common F	AATGATACGGCGACCACCGAGATCTACACTCTTTCCCTACACGACGCTCTTCCG
Deepsequence_2nd_Unique R1	CAAGCAGAAGACGGCATACGAGATCGTGATGTGACTGGAGTTCAGACGTGTGCTCTTCC G
Deepsequence_2nd_Unique R2	CAAGCAGAAGACGGCATACGAGATACATCGGTGACTGGAGTTCAGACGTGTGCTCTTCC G

Deepsequence_ 2nd_Unique R3	CAAGCAGAAGACGGCATACGAGATGCCTAAGTGACTGGAGTTCAGACGTGTGCTCTTCC G
Deepsequence_ 2nd_Unique R4	CAAGCAGAAGACGGCATACGAGATTGGTCAGTGACTGGAGTTCAGACGTGTGCTCTTCC G
Deepsequence_ 2nd_Unique R5	CAAGCAGAAGACGGCATACGAGATCACTGTGTGACTGGAGTTCAGACGTGTGCTCTTCC G
Deepsequence_ 2nd_Unique R6	CAAGCAGAAGACGGCATACGAGATATTGGCGTGACTGGAGTTCAGACGTGTGCTCTTCC G
Deepsequence_ 2nd_Unique R7	CAAGCAGAAGACGGCATACGAGATGATCTGGTGACTGGAGTTCAGACGTGTGCTCTTCC G
Deepsequence_ 2nd_Unique R8	CAAGCAGAAGACGGCATACGAGATTCAAGTGTGACTGGAGTTCAGACGTGTGCTCTTCC G
Deepsequence_ 2nd_Unique R9	CAAGCAGAAGACGGCATACGAGATCTGATCGTGACTGGAGTTCAGACGTGTGCTCTTCC G
Deepsequence_ 2nd_Unique R10	CAAGCAGAAGACGGCATACGAGATAAGCTAGTGACTGGAGTTCAGACGTGTGCTCTTCC G
Deepsequence_ 2nd_Unique R11	CAAGCAGAAGACGGCATACGAGATGTAGCCGTGACTGGAGTTCAGACGTGTGCTCTTCC G
Deepsequence_ 2nd_Unique R12	CAAGCAGAAGACGGCATACGAGATTACAAGGTGACTGGAGTTCAGACGTGTGCTCTTCC G
Deepsequence_ 2nd_Unique R13	CAAGCAGAAGACGGCATACGAGATTTGACTGTGACTGGAGTTCAGACGTGTGCTCTTCC G
Deepsequence_ 2nd_Unique R14	CAAGCAGAAGACGGCATACGAGATGGAACTGTGACTGGAGTTCAGACGTGTGCTCTTCC G
Deepsequence_ 2nd_Unique R15	CAAGCAGAAGACGGCATACGAGATTGACATGTGACTGGAGTTCAGACGTGTGCTCTTCC G
Deepsequence_ 2nd_Unique R16	CAAGCAGAAGACGGCATACGAGATGGACGGGTGACTGGAGTTCAGACGTGTGCTCTTC CG
Deepsequence_ 2nd_Unique R17	CAAGCAGAAGACGGCATACGAGATGCGGACGTGACTGGAGTTCAGACGTGTGCTCTTCC G
Deepsequence_ 2nd_Unique R18	CAAGCAGAAGACGGCATACGAGATTTTCACGTGACTGGAGTTCAGACGTGTGCTCTTCC G
Deepsequence_ 2nd_Unique R19	CAAGCAGAAGACGGCATACGAGATGGCCACGTGACTGGAGTTCAGACGTGTGCTCTTCC G
Deepsequence_ 2nd_Unique R20	CAAGCAGAAGACGGCATACGAGATCGAAACGTGACTGGAGTTCAGACGTGTGCTCTTCC G
Deepsequence_ 2nd_Unique R21	CAAGCAGAAGACGGCATACGAGATCGTACGGTGACTGGAGTTCAGACGTGTGCTCTTCC G

Deepsequence_ 2nd_Unique R22	CAAGCAGAAGACGGCATACGAGATCCACTCGTGACTGGAGTTCAGACGTGTGCTCTTCC G
Deepsequence_ 2nd_Unique R23	CAAGCAGAAGACGGCATACGAGATATCAGTGTGACTGGAGTTCAGACGTGTGCTCTTCC G
Deepsequence_ 2nd_Unique R24	CAAGCAGAAGACGGCATACGAGATAGGAATGTGACTGGAGTTCAGACGTGTGCTCTTCC G
CFTR_lib_F	CTACACGACGCTCTTCCGATCTGGAGAAATCCAGATCGATGG
CFTR_lib_R	AGACGTGTGCTCTTCCGATCTTTGAGTACAAGTATCAAATAGCAG
*Fah*_lib_F	CTACACGACGCTCTTCCGATCTagagccaatccccatttcca
Fah_lib_R	AGACGTGTGCTCTTCCGATCTTGCATGGTATCACCCCTGTA
Fah_rt-pcr_F	ttctactcttctcggcagca
Fah_rt-pcr_R	cggggagattgtggttccaa

Supplementary Note

```
function basecall(WTnuc)

files=dir('*.fastq');
for d=1:n
    filename=files(d).name;
    [header,seqs,qscore]=fastqread(filename);
    seqsLength=length(seqs);
    seqsFile=strrep(filename,'.fastq','');

    mkdir(seqsFile);
    wtLength = length(WTnuc);
    window=1:wtLength;
    sBLength=length(seqs);
    nskips=0;
    ALN=repmat('',[sBLength wtLength]);
    for i=1:sBLength

[score,alignment,start]=swalign(seqs{i},WTnuc,'Alphabet','NT');
        len=length(alignment(3,:));
        skip=0
        for j=1:len
            if (alignment(3,j)=='-'||alignment(1,j)=='-')
                skip=1;
                break
            end
            if isletter(qscore{i}(start(1)+j-1))
            else
                alignment(1,j)='N';
            end
        end
        if skip==0 && len>10
            ALN(i, start(2):(start(2)+length(alignment)-
1))=alignment(1,:);
```

```
        end
    end
     TallyNTD=zeros(5,wtLength);
     FreqNTD=zeros(4,wtLength);
     SUM=zeros(1,wtLength);
     for i=1:wtLength

TallyNTD(:,i)=[sum(ALN(:,i)=='A'),sum(ALN(:,i)=='C'),sum(ALN(:,i)
=='G'),sum(ALN(:,i)=='T'),sum(ALN(:,i)=='N')];
     end
     for i=1:wtLength
        FreqNTD(:,i)=100*TallyNTD(1:4,i)/sum(TallyNTD(1:4,i));
     end
     for i=1:wtLength
        SUM(:,i)=sum(TallyNTD(1:4,i));
     end

     save(strcat(seqsFile, '/TallyNTD'), 'TallyNTD');
     dlmwrite(strcat(seqsFile, '/TallyNTD.csv'), TallyNTD,
'precision', '%.3f', 'newline','pc');
     save(strcat(seqsFile, '/FreqNTD'), 'FreqNTD');
     dlmwrite(strcat(seqsFile, '/FreqNTD.csv'), FreqNTD,
'precision', '%.3f', 'newline','pc');
     fid = fopen('FrequencySummary.csv', 'a');
     fprintf(fid, '\n \n');
     fprintf(fid, filename);
     fprintf(fid, '\n \n');
     dlmwrite('FrequencySummary.csv', FreqNTD, 'precision',
'%.3f', 'newline', 'pc', '-append');
     dlmwrite('FrequencySummary.csv', SUM, 'precision', '%.3f',
'newline', 'pc', '-append');
end

cd(folder name')
basecall('reference sequence')
```
