# Supplementary material for: Chemical modifications of adenine base editor mRNA and guide RNA expand its application scope
Source: Nat Commun. 2020 Apr 24;11:1979. doi: 10.1038/s41467-020-15892-8 (PMC7181807; doi:10.1038/s41467-020-15892-8)
Supplement: Supplementary file 2 — Reporting Summary [file 41467_2020_15892_MOESM2_ESM.pdf]

nature research

Corresponding author(s): Wen Xue

Last updated by author(s): 03/30/2020

Reporting Summary

Nature Research wishes to improve the reproducibility of the work that we publish. This form provides structure for consistency and transparency in reporting. For further information on Nature Research policies, see Authors & Referees and the Editorial Policy Checklist .

Statistics

For all statistical analyses, confirm that the following items are present in the figure legend, table legend, main text, or Methods section.

n/a	Confirmed	
☐	☒	The exact sample size (n) for each experimental group/condition, given as a discrete number and unit of measurement
☐	☒	A statement on whether measurements were taken from distinct samples or whether the same sample was measured repeatedly
☐	☒	The statistical test(s) used AND whether they are one- or two-sided<br>*Only common tests should be described solely by name; describe more complex techniques in the Methods section.*
☒	☐	A description of all covariates tested
☒	☐	A description of any assumptions or corrections, such as tests of normality and adjustment for multiple comparisons
☐	☒	A full description of the statistical parameters including central tendency (e.g. means) or other basic estimates (e.g. regression coefficient) AND variation (e.g. standard deviation) or associated estimates of uncertainty (e.g. confidence intervals)
☐	☒	For null hypothesis testing, the test statistic (e.g. F, t, r) with confidence intervals, effect sizes, degrees of freedom and P value noted<br>*Give P values as exact values whenever suitable.*
☒	☐	For Bayesian analysis, information on the choice of priors and Markov chain Monte Carlo settings
☒	☐	For hierarchical and complex designs, identification of the appropriate level for tests and full reporting of outcomes
☒	☐	Estimates of effect sizes (e.g. Cohen's d, Pearson's r), indicating how they were calculated

Our web collection on statistics for biologists contains articles on many of the points above.

Software and code

Policy information about availability of computer code

Data collection	Illumina Miseq (software V3.1)
Data analysis	Matlab R2019a. The code was reported as Supplementary Information in this published paper: N. M. Gaudelli, A. C. Komor, H. A. Rees, M. S. Packer, A. H. Badran, D. I. Bryson, and D. R. Liu, “Programmable base editing of A • T to G • C in genomic DNA without DNA cleavage,” Nat. Publ. Gr., vol. 551, no. 7681, pp. 464–471, 2017. The script is provided as Supplementary Note.

For manuscripts utilizing custom algorithms or software that are central to the research but not yet described in published literature, software must be made available to editors/reviewers. We strongly encourage code deposition in a community repository (e.g. GitHub). See the Nature Research guidelines for submitting code & software for further information.

Data

Policy information about availability of data

All manuscripts must include a data availability statement. This statement should provide the following information, where applicable:

- Accession codes, unique identifiers, or web links for publicly available datasets
- A list of figures that have associated raw data
- A description of any restrictions on data availability

A reporting summary for this article is available as a Supplementary Information file. The source data underlying Figs. 1b-e, 2c–e, 3b, d, e and Supplementary Figs. 1a, c, e, f, 2a, b, 3a, b, 4c, d, e, 5a, c are provided as a Source Data File. The raw sequencing data have been submitted to the NCBI BioProject database (PRJNA616114 (https://www.ncbi.nlm.nih.gov/bioproject/616114) and PRJNA616118 (https://www.ncbi.nlm.nih.gov/bioproject/616118)). The All other data are available from the corresponding author upon reasonable request.

Field-specific reporting

Please select the one below that is the best fit for your research. If you are not sure, read the appropriate sections before making your selection.

- [x] Life sciences
- [ ] Behavioural & social sciences
- [ ] Ecological, evolutionary & environmental sciences

For a reference copy of the document with all sections, see nature.com/documents/nr-reporting-summary-flat.pdf

Life sciences study design

All studies must disclose on these points even when the disclosure is negative.

Sample size	2x10e5 cells were used for editing in culture system. We first performed pilot titration experiments to determine the sample size. And according to previous studies (Song et al, Nature Biomedical Engineering, 2019), we confirmed this sample size to be sufficient to ensure reproducibility. All cell samples were evaluated in at least biological triplicates (n = 3) to ensure the reproductability. For animal experiment, we described the size in the specific figure legend. The size is determined based on the availability of the mice and previous reports (Song et al, Nature Biomedical Engineering, 2019).
Data exclusions	No data was excluded.
Replication	Experiments were done in biological triplicate in culture cells, n=3 ,on different days (every three days). All attempts at replication were successful, and standard deviations were in the expected ranges.
Randomization	For all the culture-related experiments, after seeding cell into 12-well plate, we randomly decided which cells are for experiment group or control group. For mouse experiment, we randomly decide the mice treated for control or LNP.
Blinding	It is not applied to molecular and cell experiments. All mouse work are blind.

Reporting for specific materials, systems and methods

We require information from authors about some types of materials, experimental systems and methods used in many studies. Here, indicate whether each material, system or method listed is relevant to your study. If you are not sure if a list item applies to your research, read the appropriate section before selecting a response.

Materials & experimental systems

n/a	Involved in the study	
[ ]	[x]	Antibodies
[ ]	[x]	Eukaryotic cell lines
[x]	[ ]	Palaeontology
[ ]	[x]	Animals and other organisms
[x]	[ ]	Human research participants
[x]	[ ]	Clinical data

Methods

n/a	Involved in the study	
[x]	[ ]	ChIP-seq
[x]	[ ]	Flow cytometry
[x]	[ ]	MRI-based neuroimaging

Antibodies

Antibodies used	Mouse anti-Cas9 antibody (A-9000-050); Mouse anti-Fumarylacetoacetate hydrolase antibody (ab83770); mouse anti-GAPDH (EMD, MAB347); mouse anti-CFTR (UNC-596)
Validation	Mouse anti-Cas9 validated by manufacturer by western blotting against over-expressed spCas9 from HEK293T cell extract.<br>Mouse anti-Fumarylacetoacetate hydrolase antibody valisted by manufacturer by immunohistochemistry with Human liver and mouse KO tissue<br>Mouse anti-GAPDH: validated by manufacturer by western blotting from rat brain tissue lysates.<br>mouse anti-CFTR: validated by manufacturer by western blotting against CFTR protein.

Eukaryotic cell lines

Policy information about cell lines

Cell line source(s)	HEK293T, 16HBE and 16HBEge W1282X cells.
Authentication	HEK293T (ATCC) cells were valiated by supplier (ATCC) by STR analysis.16HBE (SCC150) parental cells were validated by Millipore (The supplier). 16HBEge W1282X cells were validated by Cystic Fibrosis Foundation, CFFT Lab.

Mycoplasma contamination	no
Commonly misidentified lines (See ICLAC register)	no

Animals and other organisms

Policy information about studies involving animals; ARRIVE guidelines recommended for reporting animal research

Laboratory animals	Fah mut/mut mice was a mouse model of tyrosinemia. 7weeks-old female mice were used in this study. Temperature of 65-75°F (~18-23°C) with 40-60% humidity are kept in the mouse room. A 14-hour light/10-hour dark cycle.
Wild animals	No wild animals were used in the study
Field-collected samples	No field-collected samples were used in the study
Ethics oversight	All animal study protocols were approved by the UMass IACUC.

Note that full information on the approval of the study protocol must also be provided in the manuscript.
